# Cytomegalovirus-Driven Adaptive-Like Natural Killer Cell Expansions Are Unaffected by Concurrent Chronic Hepatitis Virus Infections

**DOI:** 10.3389/fimmu.2017.00525

**Published:** 2017-05-08

**Authors:** David F. G. Malone, Sebastian Lunemann, Julia Hengst, Hans-Gustaf Ljunggren, Michael P. Manns, Johan K. Sandberg, Markus Cornberg, Heiner Wedemeyer, Niklas K. Björkström

**Affiliations:** ^1^Center for Infectious Medicine, Department of Medicine Huddinge, Karolinska Institutet, Karolinska University Hospital, Stockholm, Sweden; ^2^Department for Viral Immunology, Heinrich Pette Institute, Hamburg, Germany; ^3^Department of Gastroenterology, Hepatology and Endocrinology, Hannover Medical School, Hannover, Germany; ^4^German Center for Infection Research, Partner Site Hannover-Braunschweig, Hannover-Braunschweig, Germany

**Keywords:** natural killer cells, cytomegalovirus, killer-cell immunoglobulin-like receptor, hepatitis B virus, hepatitis C virus, hepatitis delta virus

## Abstract

Adaptive-like expansions of natural killer (NK) cell subsets are known to occur in response to human cytomegalovirus (CMV) infection. These expansions are typically made up of NKG2C^+^ NK cells with particular killer-cell immunoglobulin-like receptor (KIR) expression patterns. Such NK cell expansion patterns are also seen in patients with viral hepatitis infection. Yet, it is not known if the viral hepatitis infection promotes the appearance of such expansions or if effects are solely attributed to underlying CMV infection. In sizeable cohorts of CMV seropositive hepatitis B virus (HBV), hepatitis C virus (HCV), and hepatitis delta virus (HDV) infected patients, we analyzed NK cells for expression of NKG2A, NKG2C, CD57, and inhibitory KIRs to assess the appearance of NK cell expansions characteristic of what has been seen in CMV seropositive healthy individuals. Adaptive-like NK cell expansions observed in viral hepatitis patients were strongly associated with CMV seropositivity. The number of subjects with these expansions did not differ between CMV seropositive viral hepatitis patients and corresponding healthy controls. Hence, we conclude that adaptive-like NK cell expansions observed in HBV, HCV, and/or HDV infected individuals are not caused by the chronic hepatitis infections *per se*, but rather are a consequence of underlying CMV infection.

## Introduction

Infection with cytomegalovirus (CMV) is associated with expansion of a subset of NKG2C-expressing natural killer (NK) cells (1). Such expansions have also been observed in CMV seropositive individuals infected with other infections such as hantavirus (2), HIV-1 (3, 4), and EBV (5). Expanded NK cell populations expressing the activating NKG2C receptor, or CD2 in the case of homozygous deletion of the NKG2C gene (6), are often dominated by the expression of a single inhibitory killer-cell immunoglobulin-like receptor (KIR) receptor (7). NK cell populations with these phenotypic traits have memory-like features including the lack of adaptor proteins EAT-2 (8) and FcεR1γ (9, 10) and have distinct epigenetic characteristics compared to non-expanded NK cells (8, 10, 11).

Cytomegalovirus-driven NK cell-expansions have been reported in viral hepatitis infections (12). Furthermore, increased NKG2C-expressing NK cell populations have been observed in patients with chronic hepatitis B virus (HBV) infection (13) or hepatitis C virus (HCV) infection (14). NK cell expansions in CMV seropositive HCV-infected patients share phenotypic traits with expansions found in healthy controls including loss of the FcεR1γ adaptor (15) and superior capacity to respond to CD16 stimulation (12, 15). However, the extents to which different hepatitis virus infections contribute to or modify these NK cell expansions are unknown.

To study this, we examined NK cell expansions in HBV-, HCV-, and hepatitis delta virus (HDV)-infected patients in the context of underlying CMV infection by studying the expression of differentiation markers and inhibitory KIRs on NK cells. The results suggest that the presence of adaptive-like NK cell expansions seen in HBV, HCV, and HDV infections is primarily caused by the underlying CMV infection, and not significantly influenced by the hepatitis virus infections *per se*.

## Materials and Methods

### Patient Material

Peripheral blood mononuclear cells (PBMCs) from patients used in this study were collected at the outpatient clinic of the Department of Gastroenterology, Hepatology, and Endocrinology at Hannover Medical School in Germany. A total of 24 HBV, 18 HCV, and 28 HDV patients were analyzed, of which 20, 12, and 24 were CMV seropositive, respectively. The detailed clinical characteristics of these patients have been reported previously (16). PBMCs from 29 healthy donors, of whom 23 were CMV seropositive, were collected at the Karolinska University Hospital, Stockholm, Sweden. PBMCs were isolated through standard density-gradient separation and cryopreserved for deferred analysis.

### Monoclonal Antibodies and Viability Stains

The following monoclonal antibodies were used in the study: CD14-Horizon-V500, CD19-Horizon-V500 (BD Biosciences), CD3-PE-Cy5, CD56-ECD, CD57-PacificBlue, NKG2A-APC (Beckman Coulter), CD4-PE-Cy5, KIR3DL1-Alexa700 (Biolegend), KIR2DL1-biotin, KIR2DL3-FITC, NKG2C-PE (RnD systems), Aqua Dead Cell Stain Kit 405 nm, Strepavidin-Qdot-605, and Qdot-700 (Thermofisher).

### Flow Cytometry

Flow cytometry staining was performed as previously described (17). In brief, cryopreserved PBMCs were thawed and washed twice prior to 30 min incubation in the dark at room temperature with the desired antibody combination. Subsequently, the PBMCs were washed once and incubated for 20 min in the dark at room temperature with streptavidin-conjugates. Cells were subsequently washed twice and fixed in 2% PFA for 10 min in the dark at 4°C. Data were acquired on a BD LSRFortessa and analyzed with FlowJo software version 9.8 (LLC, OR, USA).

### CMV Serology

Cytomegalovirus IgG serology on hepatitis samples was determined at Hannover Medical School, Hannover; serology on the healthy control samples was determined at Karolinska Institutet, Sweden; both centers used Abbott ARCHITECT Anti-Cytomegalovirus IgG tests.

### Statistical Methods, Analysis Strategies, and Outlier Identification

Since expanded NK cell subsets have narrow KIR-repertoires often dominated by a single inhibitory receptor, different statistical strategies aimed at identifying outliers have previously been utilized to identify NK cell expansions (7, 18, 19). For cohorts significantly larger than ours, the Chauvenet criterion has been applied to select for NK cell expansions combined with cellular maturation (7, 18). However, along with our smaller cohorts, the lower expression of some KIRs during hepatitis infections may affect selection. In line with how we previously identified NK cell expansions (19), a variation of the Tukey’s range test accounting for the cohort size was utilized to identify potential NK cell expansions. In more detail, CD56^dim^ NK cells were subdivided into subsets based upon their expression of NKG2A and NKG2C. Subsequently, the expression repertoire of KIR2DL1, KIR2DL3, and KIR3DL1 was assessed. Particular KIR-defined subpopulations were considered as expanded populations if their population frequency was greater than one interquartile range above quartile three in each cohort. Threshold population sizes of either 5 or 20% were both analyzed. Further ensuring that the particular KIR-expressing NK cell subpopulation was a substantial constituent of the NK cell subset (e.g., NKG2A^−^NKG2C^+^), it was required to be greater than quartile one of the NK cell subset. Finally the geometric MFI of CD57 on the KIR-expressing NK cell subpopulation had to be greater than the geometric MFI of the subject’s total CD56^dim^ NK cells, ensuring that the identified outliers contained terminally differentiated NK cells (7, 20). Identifications of NK cell expansions were performed using Microsoft Excel version 14.4.3 (Microsoft Corporation, Washington, USA). Data were analyzed using Prism version 5.0d (GraphPad Software Inc., CA, USA). For the discrete statistics, non-parametric tests were used as outlined in the respective figure legends.

## Results

### Validation of CMV-Associated Alterations in NK Cell Differentiation Status

**Figure 1:**
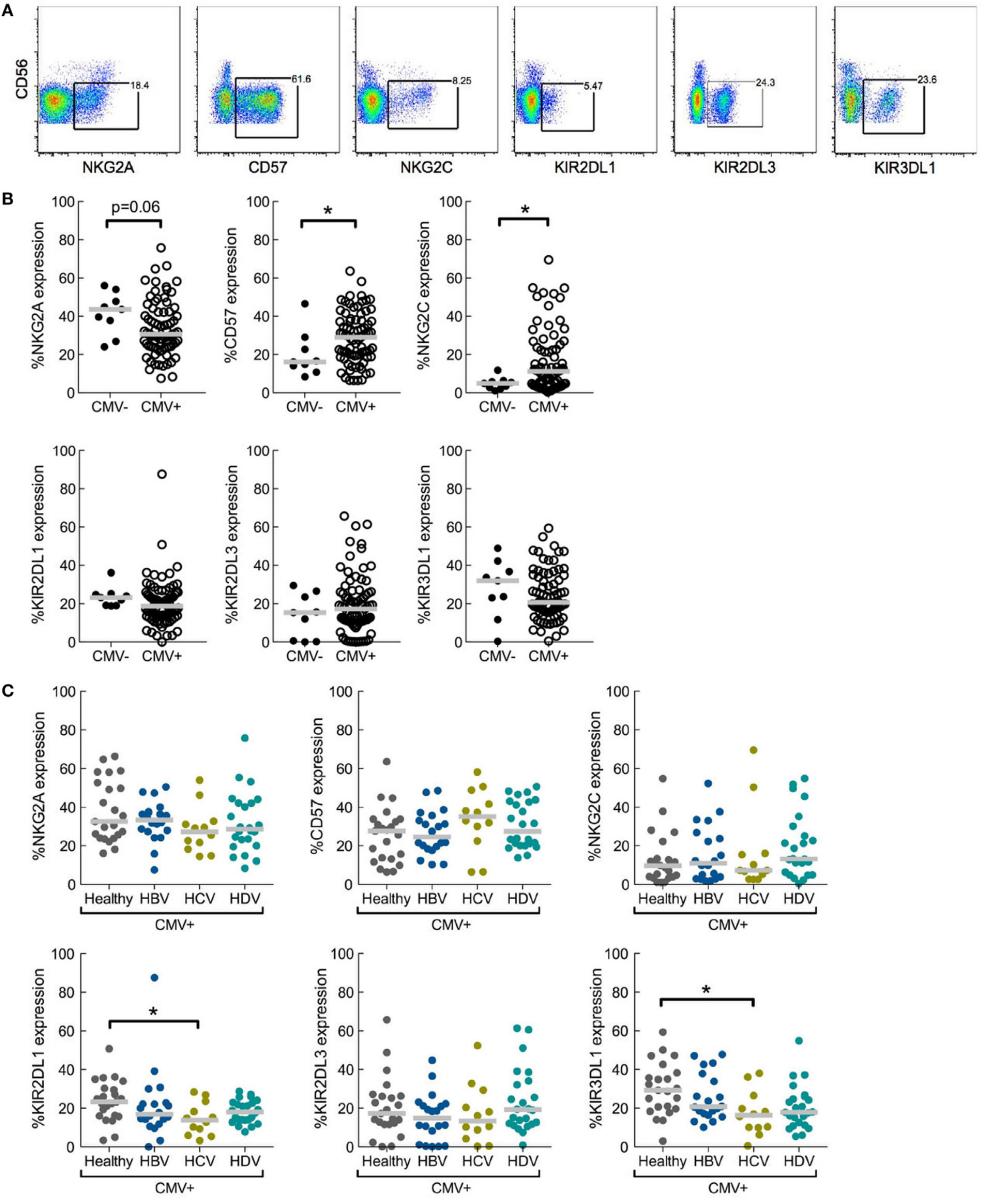
**Role of underlying cytomegalovirus (CMV) infection in chronic viral hepatitis infections with respect to effects of natural killer (NK) cell differentiation and killer-cell immunoglobulin-like receptor (KIR) expression**. **(A)** Representative staining of differentiation-associated receptors on CD56^dim^ NK cells. Data shown from total lymphocytes have CD3, CD4, CD14, CD19, and dead cell marker positive cells excluded. **(B)** Expression of NKG2A, CD57, NKG2C, KIR2DL1, KIR2DL3, and KIR3DL1 on CD56^dim^ NK cells in CMV seronegative (CMV^−^) or seropositive (CMV^+^) individuals, irrespective of hepatitis status; CMV^−^ *n* = 9 and CMV^+^ *n* = 79. Statistical analysis performed with Mann–Whitney test. **(C)** Expression of NKG2A, CD57, NKG2C, KIR2DL1, KIR2DL3, and KIR3DL1 on CD56^dim^ NK cells in CMV seropositive individuals split into healthy (*n* = 23) and hepatitis virus infected [*n* = 20, 12, and 24 for hepatitis B virus (HBV), hepatitis C virus (HCV), and hepatitis delta virus (HDV), respectively]. Statistical analysis performed with Kruskal–Wallis test and Dunn’s multiple comparison. Gray bar indicates median. *p < 0.05.

First, NK cell differentiation-associated receptors NKG2A, CD57, and NKG2C, as well as inhibitory receptors KIR2DL1, KIR2DL3, and KIR3DL1 were stained for on CD56^dim^ NK cells (Figure 1A). Expression of these markers was assessed in relation to CMV serostatus in the entire study cohort. As expected from previous reports (1, 21), CMV infection was associated with a higher frequency of CD56^dim^ NK cells expressing NKG2C and CD57, and with lower NKG2A expression compared to CMV seronegative individuals. However, CMV serostatus did not significantly alter expression of individual KIRs (Figure 1B).

### Viral Hepatitis Does Not Alter NK Cell Differentiation as Assessed in CMV Seropositive Patients and Controls

Of the 88 individuals assessed in the entire study cohort, 79 were confirmed CMV seropositive permitting us to study the influence of hepatitis infection on a CMV seropositive background in relation to NK cell differentiation (Figure 1C). No major differences in expression of NKG2A, NKG2C, or CD57 were observed when comparing CD56^dim^ NK cells in CMV seropositive chronic hepatitis patients with corresponding CMV seropositive healthy controls (Figure 1C).

A recently published study including the same patient cohorts indicated that there was no overall difference in the expression of inhibitory KIRs on NK cells during hepatitis infection compared to healthy controls (16). However, in that report expression patterns of individual KIRs were not assessed. Here, a more detailed examination was performed. NK cells from CMV seropositive HCV-infected patients showed a significant decrease in expression of KIR2DL1 and KIR3DL1 relative to healthy controls, while expression of KIR2DL3 was unaltered (Figure 1C). HBV infection and HDV infection had no significant impact on KIR expression (Figure 1C).

### Presence of CMV-Driven NK Cell Expansions Remain Unaltered in Chronic Viral Hepatitis

**Figure 2:**
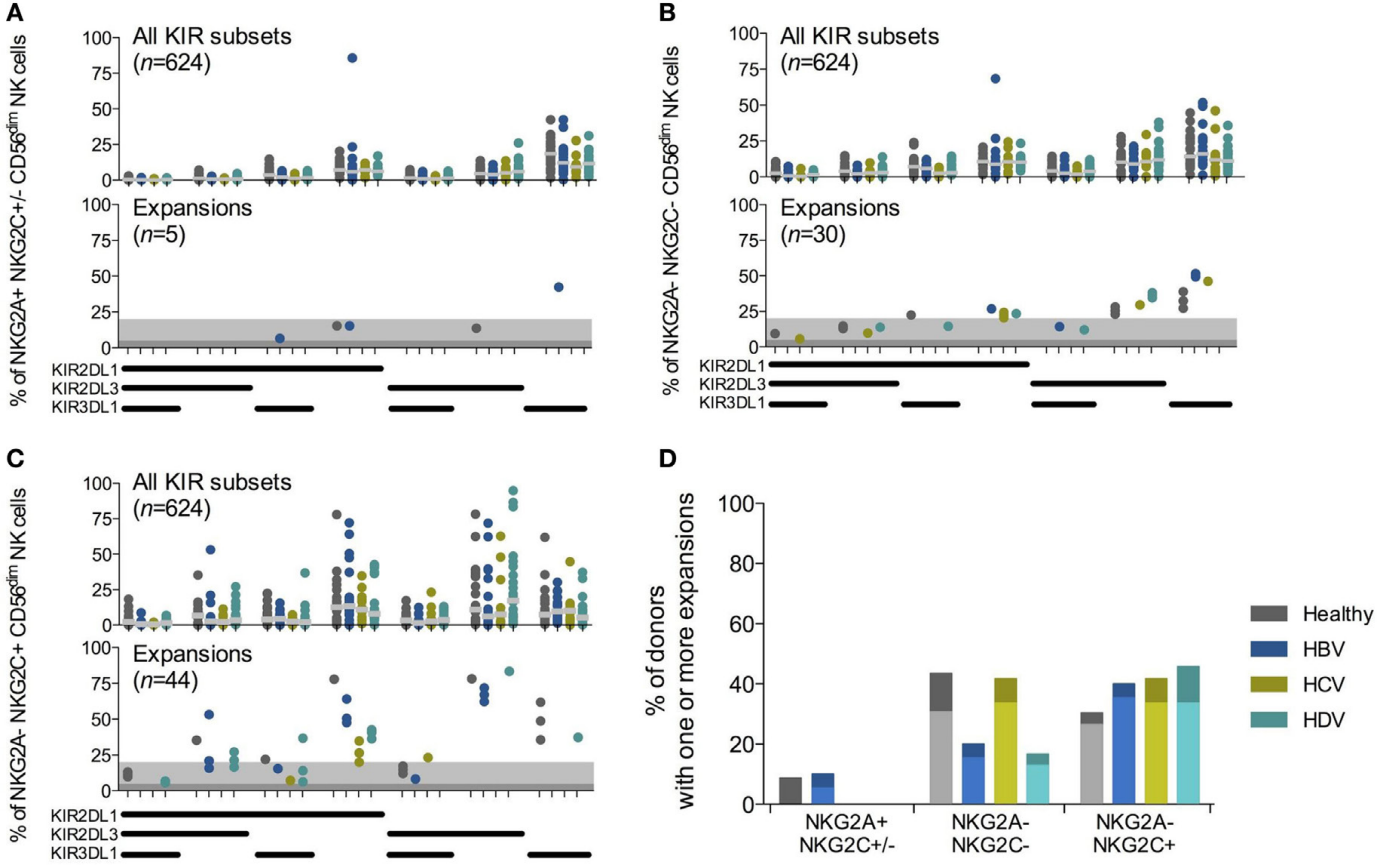
**Chronic viral hepatitis does not affect the frequency of cytomegalovirus (CMV)-driven natural killer (NK) cell expansions**. **(A–C)** Killer-cell immunoglobulin-like receptor (KIR)-repertoire and adaptive-like NK cell expansions within NKG2A^+^NKG2C^+/−^ **(A)**, NKG2A^−^NKG2C^−^ **(B)**, and NKG2A^−^NKG2C^+^ **(C)** CD56^dim^ NK cell populations in CMV seropositive individuals. Upper-row figures display all KIR subpopulations [*n* = 23, 20, 12, and 24 for healthy individuals and hepatitis B virus (HBV), hepatitis C virus (HCV), and hepatitis delta virus (HDV) infected individuals, respectively]. Gray bar indicates median. Bottom-row figures display the identified expansions. The dark gray shading indicates a 5% and the light gray shading a 20% threshold for confirmation of expansions. **(D)** Percentage of CMV seropositive individuals that have at least one measured expansion within the CD56^dim^ NK cell population. Twenty percent threshold results depicted in light colored bars. Chi-squared test for all NK cell subsets: healthy, *p* < 0.02; HBV, *p* < 0.04; HCV, *p* < 0.08; HDV, *p* < 0.01; and between NKG2A^−^ subsets: healthy, *p* = 1; HBV, *p* = 0.27; HCV, *p* = 1; HDV, *p* < 0.17. Five percent threshold results depicted as light and dark bars, Chi-squared test for all NK subsets: healthy, *p* < 0.03; HBV, *p* < 0.074; HCV, *p* < 0.03; HDV, *p* = 0.0004; and between NKG2A^−^ subsets: healthy, *p* < 0.55; HBV, *p* < 0.3; HCV, *p* = 1; HDV, *p* < 0.06.

As described, expansions of NKG2C^+^ NK cells with a specific KIR-repertoire driven by CMV infection have been observed in chronic hepatitis virus infections (12). However, whether hepatitis virus infection affects the prevalence of these expansions is unknown. Adaptive-like NK cell expansions have previously been identified based on deviations in KIR expression profiles, using statistical strategies such as the Chauvenet criterion (7, 18) and the Tukey’s range test (19), combined with cellular differentiation. As our analysis included patients with different hepatitis infections, likely affecting receptor expression to varying degrees, each group was analyzed separately. NK cell subsets were assessed for the presence of expansions (see Materials and Methods) and quantified (Figures 2A–C). For both NKG2A^−^NKG2C^−^ and NKG2A^−^NKG2C^+^ NK cell subsets, there was no overall difference in the number of CMV seropositive individuals with NK cell expansions, irrespective of chronic hepatitis virus infections (Figure 2D). Since only 9 out of 88 studied patients were CMV seronegative, it was not possible to determine the influence of chronic viral hepatitis on potential expansions within this cohort. Together, we conclude that there is no difference between the prevalence of adaptive-like NK cell expansions observed in CMV seropositive subjects with HBV, HCV, and HDV infection as compared to CMV seropositive healthy controls.

## Discussion

Adaptive-like expansions of certain NK cell subsets are known to occur in response to human CMV infection. As described, such expansions are typically made up of NKG2C^+^ NK cells with specific KIR expression patterns. Such expansions have been observed in several viral infections including viral hepatitis infections (12, 15). Yet, it is not known if viral hepatitis infection as such contributes to or promotes the appearance of such expansions, or if effects are primarily due to underlying CMV infection. Utilizing a CMV seropositive patient cohort with different forms of chronic viral hepatitis (HBV, HCV, and HDV), we confirmed the existence of large populations of adaptive-like CD57 expressing NK cells. However, the presence of these expansions was largely unrelated to hepatitis status. Since the vast majority of studied subjects were CMV seropositive, it was not possible to determine the influence of chronic viral hepatitis infections on potential adaptive-like NK cell expansions in CMV seronegative individuals. Based on results obtained in this study, we conclude that the observed expansions in hepatitis virus infected individuals (12) are attributed largely, if not exclusively, to underlying CMV infection.

Except for expression of NKG2C combined with a narrow KIR-repertoire, adaptive-like NK cell expansions have also been reported to express high levels of CD57, LILRB1, and CD2 while largely lacking NKG2A, NKp30, CD161, EAT-2, and FcεR1γ expression (2, 6–8). Although the adaptive NK cells display unique functional characteristics (2, 6–8), the phenotypic diversity within adaptive-like expanded subsets appears lower as compared to, e.g., the conventional CD56^dim^ NK cell subset (22, 23). It is currently unclear to which the degree the phenotype of adaptive-like NK cell expansions is affected in settings of disease, e.g., during chronic viral infections. In this study, we utilized expression of NKG2C, KIRs, and CD57 combined with absence of NKG2A to identify adaptive-like NK cell expansions. We could not detect any differences in expression of NKG2A, CD57, and NKG2C when comparing patients with controls. However, it is still plausible that other phenotypic traits associated with adaptive-like expansions are uniquely affected during chronic viral hepatitis.

It has been previously shown that KIR2DL2/3 is the most frequently expressed KIR on expanded adaptive-like NK cells during chronic hepatitis virus infections (12). While, we were unable to *KIR* genotype the present patient cohort, to allow us to precisely examine KIR2DL2 expression, we did observe maintenance of KIR2DL3 expression and down-regulation of KIR2DL1 and KIR3DL1 in chronic hepatitis C compared to healthy controls (Figure 1C). This is in line with previous results from other groups (13, 24). Preservation of expression of KIR2DL3 may be due either to immune adaptation or selective pressure in response to hepatitis infection. Intriguingly, it was recently shown that HCV sequence variation in core protein-derived HLA-presented epitopes alters the binding of KIR2DL3 to its HLA-ligand with functional consequences for NK cells suggesting a potential pathway for viral escape via KIR2DL3 (25).

There are contradictory results regarding NKG2C expression on NK cells during hepatitis infections. A systematic review suggested increased levels of NKG2C on CD56^dim^ NK cells during HBV infections relative to healthy donors (26). Further, levels of NKG2C in HBV infections were shown to be greater than those in HCV infections (13), while others have seen increased NKG2C during HCV infection (24). Data from our cohorts do not confirm these reports. As CMV serostatus associates with NKG2C expression, it is possible that these reported discrepancies would disappear had CMV serological status been considered. Indeed, such bias inferred by CMV was previously reported for individuals infected with HIV-1 (4).

In this study, we employed a non-parametric method to identify expanded NK cell populations. In brief, CD56^dim^ NK cells were separated into subsets based on expression of NKG2A and NKG2C, further subdivided based upon their expression profile of KIR2DL1, KIR2DL3, and KIR3DL1, and assessed for KIR-expressing NK cell expansions. NK cell expansions were in all assessed patient groups and healthy controls primarily confined within the NKG2A^−^NKG2C^+^ or the NKG2A^−^NKG2C^−^ groups of CD56^dim^ NK cells (Figures 2A–C). Few, if any, expansions had an NKG2A^+^ phenotype (Figure 2A). Although NK cell expansions have predominantly been described as NKG2C^+^ (18), NKG2C^−^ CD2^+^ NK cell expansions have also been reported (6). Therefore, we speculate that the NKG2A^−^NKG2C^−^ NK cell expansions we observed may have had another activating receptor associated with their function, e.g., CD2 or activating KIRs. Taken together, the results validated our statistical strategy for identification of expansions, permitting us to compare how common expanded NK cell subsets were in CMV seropositive individuals with chronic viral hepatitis infections compared to healthy controls.

The NK cell expansions that we identified may be the reported adaptive-like NK cells, which lack the adaptor protein FcεR1γ and have been associated with reduced liver damage in HCV infection (15). Thus, the role for such cells, with increased levels of IFN-γ production in response to CD16 stimulation (10), should be thoroughly assessed in acquisition and progression of hepatitis virus infections in future studies.

Taken together, these results suggest that the NK cell differentiation status and the presence of NK cells with an adaptive phenotype, when controlled for CMV, remain unaffected by chronic viral hepatitis infection.

## Ethics Statement

This study was carried out in accordance with the recommendations of the Ethics Committee of Hannover Medical School, Hannover, Germany with written informed consent from all subjects. All subjects gave written informed consent in accordance with the Declaration of Helsinki. The protocol was approved by the Ethics Committee of Hannover Medical School, Hannover, Germany.

## Author Contributions

DM performed most of the experiments, contributed to study design, acquisition of data, analysis, and drafting of the manuscript; SL performed most of the experiments, contributed to study design, and acquisition of data; JH contributed to study design and analysis; MM, JS, and MC contributed to study design and data interpretation; H-GL and HW contributed to study design, data interpretation, drafting the manuscript, and supervision of the work; NB contributed to study design, data analysis, drafting of the manuscript, and supervised the work.

## Conflict of Interest Statement

The authors declare that the research was conducted in the absence of any commercial or financial relationships that could be construed as a potential conflict of interest.

## Abbreviations

CMV, cytomegalovirus; HBV, hepatitis B virus; HCV, hepatitis C virus; HDV, hepatitis delta virus; KIR, killer-cell immunoglobulin-like receptor; NK, natural killer; PBMC, peripheral blood mononuclear cell.
